# Validation and meaningful within-patient change in work productivity and activity impairment questionnaire (WPAI) for episodic or chronic migraine

**DOI:** 10.1186/s41687-023-00552-4

**Published:** 2023-04-04

**Authors:** Janet H. Ford, Wenyu Ye, David W. Ayer, Xiaojuan Mi, Swati Bhandari, Dawn C. Buse, Richard B. Lipton

**Affiliations:** 1 Eli Lilly and Company, 893 Delaware St, Indianapolis, IN 46225 USA; 2 TechData Services Company, King of Prussia, PA USA; 3 Department of Neurology, Albert Einstein College of Medicine, Bronx, NY USA; 4 Headache Center, Montefiore Medical Center, Bronx, NY USA

**Keywords:** Absenteeism, Non-work-related activity impairment, Chronic migraine, Episodic migraine, Meaningful change, Patient-reported outcome, Presenteeism, Validation, Work Productivity and Activity Impairment questionnaire (WPAI), Work productivity loss

## Abstract

**Background:**

No available studies demonstrate validity and meaningful change thresholds of Work Productivity and Activity Impairment (WPAI) questionnaire in patients with migraine. In this post-hoc analysis, we assessed reliability, validity, responsiveness, and meaningful within-patient change from baseline to Month 3 for Work Productivity and Activity Impairment (WPAI) domain scores in patients with episodic migraine (EM) or chronic migraine (CM).

**Method:**

The Phase 3, multicenter, randomized, double-blind, placebo-controlled CONQUER study (NCT03559257, N = 462) enrolled patients with EM or CM who failed two to four categories of prior preventive medication in past ten years. The analyses were performed for WPAI domain scores (absenteeism, presenteeism, overall work productivity, and non-work-related activity impairment). Migraine Specific Quality of Life Questionnaire version 2.1 (MSQv2.1) domain scores (Role Function-Restrictive [RFR] and Role Function-Preventive [RFP]), and monthly migraine headache days were used as anchors. Responder criteria were changes from baseline to Month 3 for each of these anchors and were defined as: increase in MSQ-RFR by ≥ 25.71 points and MSQ-RFP by ≥ 20.00 points and a 50% reduction in monthly migraine headache days. Assessments were performed for overall population, and patients with EM or CM. The meaningful change threshold was determined based on Youden index, Phi coefficient and sensitivity.

**Results:**

Of 462 randomized patients, 444 who completed WPAI questionnaire were included in post-hoc analysis. Test–retest reliability over 3 months in a stable subgroup revealed moderate correlations for non-work-related Activity Impairment (ICC = 0.446) presenteeism (ICC = 0.438) and a fair correlation for overall work productivity loss (ICC = 0.360). At baseline, all correlations between WPAI domain scores and continuous anchor variables exceeded recommended threshold of ≥ 0.30, except for WPAI domain scores with number of monthly migraine headache days. Patients achieving pre-specified responsiveness thresholds for monthly migraine headache days, and MSQ-RFP, MSQ-RFR from baseline to Month 3 (responders) showed significant improvements in WPAI domain scores compared with non-responders (*P* < 0.001). The meaningful change thresholds of -20 (% unit) were identified for WPAI domain scores.

**Conclusion:**

In conclusion, WPAI has sufficient validity, reliability, responsiveness, and appropriate interpretation standards to assess the impact of EM or CM on presenteeism and overall work productivity loss and non-work-related activity impairment.

***Trial registration*:**

NCT number of CONQUER study, NCT03559257.

**Supplementary Information:**

The online version contains supplementary material available at 10.1186/s41687-023-00552-4.

## Introduction

The global prevalence of migraine varies among studies, a recent update provides a summary estimate of 14% and shows a prevalence peak in mid-life [1–5]. A review addressing the impact of episodic migraine (EM) and chronic migraine (CM) on employment absenteeism and presenteeism indicated that patients with migraine lost an average of 4.4 working days and experienced reduced work productivity for an additional 11.4 days annually [6]. A reduction in work productivity has implications for people with migraine, families, employers, and society.

A recent global survey, “My Migraine Voice”, evaluated the social and economic impact of migraine in patients with ≥ 4 monthly migraine days who were receiving preventive treatment for EM or CM [7]. The findings of the survey indicated that an increase in number of migraine preventive treatment failures was associated with increased rates of absenteeism (work hours missed from total work hours), presenteeism (degree of productivity affected while working), work productivity (overall work productivity affected), and non-activity-related activity impairment (degree of productivity affected in regular unpaid activity) as measured by the Work Productivity and Activity Impairment (WPAI) instrument [7]. These cross-sectional results from “My Migraine Voice” most likely reflect confounding by indication, that is, patients with more severe disease are more likely to receive treatment.

Guidelines for clinical trials of migraine treatments recommend the use of Patient Reported Outcome (PRO) measures [8, 9]. Specifically, Guidelines of the International Headache Society (IHS) for controlled trials of preventive treatment of EM and CM suggest measuring the mean change from baseline in the WPAI questionnaire [8, 9]. The WPAI is a PRO measure that assesses the impact of health problems on work-related productivity and non-work-related activity impairment over the past 7 days [10, 11]. Meaningful change (improvement) thresholds for the WPAI may be useful for clinical interpretation of the impact of a disease on patients’ ability to work [12]. The reliability, validity, and responsiveness of WPAI in patients across various disease areas have been previously reported [13–17]. Additionally, content validity of WPAI is also established in previous publications and it is recommended by IHS [9, 18, 19]. Qualitative research has confirmed the conceptual importance of work productivity for people with migraine, and several real-world and population-based studies have utilized the WPAI questionnaire to assess the impact of migraine on work productivity [20–28]. However, to the best of our knowledge, there is no report determining the validity and meaningful change of WPAI in patients with migraine.

Recently, the CONQUER study demonstrated the efficacy and safety of galcanezumab versus placebo in patients with EM or CM who had previously failed to benefit from or tolerate two to four standard-of-care categories of migraine preventive medications [29]. Significantly greater reductions (*p* < 0·0001) in monthly migraine-headache days averaged across Months 1 to 3 were reported in patients treated with galcanezumab versus placebo. Furthermore, reduction in WPAI domain scores from baseline were significantly greater (*p* ≤ 0.0004) in the galcanezumab group compared with placebo [30]. In this post-hoc analysis of the CONQUER study, we aim to assess the reliability, validity, responsiveness, and meaningful within-patient change from baseline to Month 3 for the WPAI domain scores in patients with EM or CM.

## Methods

### Study design and patient population

CONQUER (NCT03559257, N = 462) was a Phase 3, multicenter, randomized, double-blind, placebo-controlled study. The details on the study design and patient population have been previously published [29]. In brief, patients meeting the International Classification of Headache Disorders, third edition (ICHD-3) [31, 32] criteria for EM or CM and who failed two to four categories of prior preventive medications in the past ten years due to lack of efficacy (after at least two months at maximum tolerated dose) or safety and tolerability reasons or both were included. Patients were required to have four or more monthly migraine-headache days and one or more monthly headache-free days on average during the three months prior to screening. Eligible patients were randomly assigned (1:1) to monthly subcutaneous injections of either galcanezumab 120 mg (loading dose 240 mg) or placebo administered by subcutaneous injection once monthly over 3-month double-blind phase. All patients received galcanezumab 120 mg during 3-month open-label phase. The study protocol was approved by the Institutional Review Board, Medical Ethics Committee, or Medical Research and Ethics Committee of the participating study sites. The study was conducted in concordance with the Declaration of Helsinki guidelines. All patients provided written informed consent before participation in CONQUER trial.

Subgroup analyses were conducted based on EM and CM status as defined in the clinical trial protocol based on the baseline monthly migraine-headache days. CM was defined as ≥ 8 migraine headache days, with ≥ 15 headache days (migraine or nonmigraine). EM was defined as not meeting the criteria for CM and having at least 4 migraine headache days.

Secondary objectives of the CONQUER study, among others, were to compare galcanezumab with placebo on changes in disability and quality of life using PRO measures. The objectives of this post-hoc analyses were to assess the reliability, validity, responsiveness and determine meaningful within-patient change thresholds for the WPAI domain scores.

### Study measures

The WPAI-Specific Health Problem version 2 is a six-item questionnaire, with a recall period of past seven days. The WPAI questionnaire was collected at the baseline and at the Month 3 visit during double-blind period to assess past one week WPAI score. The instrument includes questions on employment status, hours missed from work due to the specific health problem (i.e. migraine), hours missed from work for other reasons, hours worked, the degree that migraine affected productivity while working, and the degree that migraine affected other daily activities [33]. Based on the responses to the six items, four scores for absenteeism, presenteeism, overall work productivity, and non-work-related activity impairment were derived as indicated in the Statistical Analysis section. The percentages were calculated as amount of time lost in each domain divided by the amount of time in each domain.

Details on calculation of WPAI scores are provided in Additional file 1. All WPAI scores were presented as percentage units across this paper.

The Migraine Specific Quality of Life Questionnaire version 2.1 (MSQv2.1) is a self-administered disease specific quality of life questionnaire [34]. The recall period for MSQ is the past 4 weeks. The MSQ score data was collected at baseline and month 3 visit during double-blind phase. The instrument consists of 14 items that assess 3 domains: (1) Role Function-Restrictive (RFR); (2) Role Function-Preventive (RFP); and (3) Emotional Function. This study focused only on MSQ RFR and RFP domains. The MSQ domain scores range from 0 (worst functional health status) to 100 (best functional health status). Increasing scores indicate improvements in quality of life [35].

Patients enrolled in the study used an electronic daily diary to record headache features and use of acute headache medications throughout the study. The number of monthly migraine headache days (per 30-day period) was the primary measure in the CONQUER study. A migraine headache day was defined as a calendar day on which a migraine or probable migraine occurred. Migraine was defined as a headache, with or without aura, of ≥ 30 min duration with both of the following required features (A and B):

A. At least 2 of the following headache characteristics: unilateral location, pulsating quality, moderate or severe pain intensity, aggravation by or causing avoidance of routine physical activity, AND
B. During headache at least one of the following: nausea and/or vomiting, photophobia and phonophobia (definition adapted from IHS ICHD-3). Probable migraine headache was defined as a headache of ≥ 30 min duration, with or without aura, but missing one of the migraine features in the IHS ICHD-3 definition [32].

In calculating the number of migraine headache days for each period, if the period was not equal to 30 days, the number of migraine headache days was adjusted by multiplying the number of migraine headache days by (30/x) where ‘x’ was the total number of non-missing diary days in the period assuming that the proportion of days with migraine is the same for days with missing and non-missing electronic patient-reported outcomes (ePRO). If more than half ePRO diary days are missing, monthly migraine headache days for that 1-month period were considered missing. Additional patient-reported data were recorded directly by the patient on an electronic tablet at the time of clinic visits.

MSQ-RFR, MSQ-RFP, and monthly migraine headache days were selected as independent anchors for this study. We have not included the Patient Global Impression of Severity (PGI-S) and Migraine Disability Assessment (MIDAS) as anchors because of lower Youden index observed in the analysis for both anchors, (Youden index < 0.25) and bimodal trends in the Youden index plots. Established responder criteria consisted of changes from baseline to Month 3 for each of these anchors, and were defined as: an improvement in MSQ-RFR by ≥ 25.71 points, an improvement in MSQ-RFP by ≥ 20.00 points [36], a 50% reduction in monthly migraine headache days.[37], while the non-responder were defined as: a < 25.71 points improvement or stay the same or worsening on MSQ-RFR score, a < 20 improvement or stay the same or worsening on the MSQ-RFP score [33], a < 50% reduction in monthly migraine headache days [9, 34, 38]. Patients who did not meet the responder criteria were defined as non-responders for each of the anchors. Responder and non-responder status was separately defined for each anchor.

### Statistical analysis

A statistical analysis plan was developed prior to the execution of this post-hoc analysis. In this post-hoc analysis, reliability, validity, responsiveness (ability to detect change), and meaningful within-patient change in WPAI score were assessed before the use of the 3 anchor-based responder definitions indicated above. WPAI absenteeism, presenteeism, overall work productivity, and non-work-related activity impairment scores are calculated from the responses of the 6 items of the WPAI (employment status, hours missed from work due to migraine, hours missed from work for other reasons, hours actually worked, degree migraine affected productivity while working and degree migraine affected productivity in regular unpaid activities) as impairment percentages [33]. Higher numbers indicate greater impairment and less productivity (i.e., worse outcomes). Calculations are completed for individuals who responded that they were employed (Additional file 1). WPAI scores were presented as percentage. Assessments were performed for the overall population, and patients with EM or CM. Descriptive statistics (including mean, standard deviation, and floor and ceiling effects) were used to determine the distribution of scores for WPAI. As the scores and changes from baseline to Month 3 for WPAI absenteeism, and correlations between WPAI absenteeism and selected anchor of monthly migraine headache days were very low in the patient population, it was determined during the initial stage of this analysis that it was not appropriate to evaluate responsiveness for this measure alone (i.e. without accounting for presenteeism). Therefore, the analyses were performed for three WPAI domain scores- presenteeism, overall work productivity loss (absenteeism plus presenteeism), and non-work-related activity impairment using the selected anchors (MSQ-RFR, MSQ-RFP, and monthly migraine headache days).

The methods for anchor selection included evaluating Spearman’s correlation analyses to test hypotheses about relationships between outcome variables at baseline (overall, and EM/CM subpopulations). Correlations between outcomes were determined using the non-parametric Spearman’s rank correlation coefficient (Rho) because the data is not assumed to be normally distributed. Multiple outcomes were evaluated, and heat maps were created by plotting Spearman’s Rank Correlation Coefficients (Rho) between these outcomes and the WPAI. Absolute value of Rho > 0.90 to ≤ 1.00 indicated very high correlation, ≥ 0.70 to ≤ 0.90 indicated high correlation, ≥ 0.60 to < 0.70 indicated moderate high correlation, ≥ 0.50 to < 0.6 indicated moderate correlation, ≥ 0.40 to < 0.50 indicated moderate low correlation, ≥ 0.30 to < 0.40 indicated low correlation, and 0.00 to ≤ 0.30 indicated negligible correlation. These methods resulted in the selected anchors, as the other outcomes had weaker associations and were not suitable anchors, such as the MSQ Emotional Domain [39, 40].

#### Reliability

Test–retest reliability was assessed in a population considered to be stable. This group received placebo and had a change of one day or less in their number of migraine-headache days per month. WPAI domain scores at baseline and Months 3 were used to assess test–retest reliability. Intra-class correlation coefficients (ICC) were calculated using the two-level random-intercept-only mixed model [41].

$$y_{ij} = \beta_{0} + \mu_{0j} + e_{ij} ,$$

where $y_{ij}$ is the WPAI score for subject *i* and time *j;* the random intercepts $\mu_{0j}$ have variance ${\upsigma }_{u0}^{2}$ and the residuals $e_{ij}$ have variance ${\upsigma }_{e}^{2}$. The ICC is the ratio of the random intercept variance to the total variance$:$

$$ICC = \frac{{\sigma_{u0}^{2} }}{{\sigma_{u0}^{2} + \sigma_{e}^{2} }}.$$

The ICC values were classified as: 0.01 to 0.20 (slightly fair), 0.21 to 0.40 (fair), 0.41 to 0.60 (moderate), 0.61 to 0.80 (substantial), and 0.81 to 1.00 (almost perfect agreement) [42].

#### Validity

Construct validity of WPAI domain scores with MSQ-RFR, MSQ-RFP, and monthly migraine-headache days was assessed using Spearman’s correlation analyses at baseline. The Spearman’s rank correlation coefficient measures the strength and direction of association between two variables using a non-parametric correlation statistic with values ranging from -1 to + 1. We defined seven levels of correlation between outcomes based on the absolute values of Rho: 0.70–1.00 (high), 0.60–0.70 (moderate high), 0.50–0.60 (moderate), 0.40–0.50 (moderate low), 0.30–0.40 (low), 0.20–0.30 (negligible), and 0–0.20 (negligible-low).

#### Responsiveness

Anchor-based responder analyses were performed to assess the ability to detect change in WPAI domain scores by responder status for each of the three anchors (MSQ-RFR, MSQ-RFP, and monthly migraine-headache days). The least square mean changes from baseline to Month 3 for WPAI for both responders and non-responders for each of the anchor variables was estimated and compared. Statistically significant differences (*p* < 0.05) in change in WPAI domain scores between responders and non-responders of anchor variables indicated responsiveness or ability to detect change in the WPAI domain scores.

Analysis of covariance (ANCOVA) models were utilized to examine differences in the change in WPAI domain scores from baseline to Month 3 among patients in all the anchor-based responder groups; the assessment evaluates the past 7-days at both time points. The change in WPAI presenteeism, overall productivity loss, and non-work-related activity impairment was the dependent variable, while the responder group variable was included as the independent variable in the model. Baseline WPAI presenteeism, overall productivity loss, and non-work-related activity impairment was adjusted for the respective models.

#### Meaningful within-patient change thresholds

Anchor-based analyses were used to determine the meaningful within-patient change in WPAI domain scores. Monthly migraine headache days decreased by 50%, MSQ-RFR scores decreased by ≥ 25.71 points and MSQ-RFP scores decreased by ≤ 20 points were selected as anchors.

WPAI change score threshold was estimated by logistic regression with the selected anchor as a dependent variable and change from baseline WPAI score as an independent variable. Discriminative ability between the responder and non-responder groups were assessed using receiver-operator characteristic (ROC) curves, Youden index (YI), Phi correlations, and Concordance (C) statistics, which is the equivalent of the area-under-curve (AUC) of a ROC curve.

The YI, a frequently used summary measure in ROC analyses, measures the effectiveness of a predictive marker, and enables selection of an optimal threshold value for that marker. The YI was calculated at each level of improvement in the WPAI (c): [YI (c) = sensitivity (c) + (specificity (c) −1)]. Its value ranges from 0 through 1; higher value indicates better discriminative ability between the responders and non-responders. The optimal cut-score is identified as the point at which YI is maximal in the ROC analysis [43]. A threshold of change from baseline WPAI score was chosen so that YI was high for all anchor variables. The phi coefficient is a measure of association for two binary variables, and it was calculated at each level of improvement in the WPAI. The AUC has values that range from 0.5 to 1.0. AUC values closer to 0.5 indicated that the accuracy of model at predicting outcome is based on chance. Values from 0.7 to 0.8 indicate a good model. Values of 0.8 to 0.9 indicate excellent discrimination, and ≥ 0.9 show outstanding discrimination between responder and non-responder groups [44]. Empirical cumulative distribution function (eCDF) plots were generated to display the change scores from baseline to Month 3 by anchor responder groups and to provide supporting evidence for the optimal threshold. All analyses were conducted based on a 2-sided significance level of 0.05 and using SAS software Version 9.4 (SAS institute, Cary, NC).

## Results

### Baseline demographic and disease characteristics

A total of 462 patients were randomized and received at least one dose of the study drug in the CONQUER study; 444 patients who completed the WPAI questionnaire for non-work-related activity impairment were included in post-hoc analyses (EM, n = 261; CM, n = 183). Of the 444 patients, overall work productivity loss data were collected from 315 patients with employment (complete data: 289; missing data: 26). Presenteeism data were collected for 315 patients (completed data: 284; missing data: 31). This analysis used complete data for measurements of WPAI at both baseline and month 3.

Baseline demographics and disease characteristics are presented in Table 1. For the overall population providing data on the WPAI, mean (SD) age was 45.9 (11.81) years, and the majority of patients (n = 381, 85.8%) were female. The WPAI absenteeism, presenteeism, overall work productivity loss, and non-work-related activity impairment scores presented as mean (SD) were 9.37 (17.85), 42.75 (23.24), 46.86 (25.69), and 50.97 (23.91), respectively. Baseline demographics and disease characteristics of employed patients were similar to those of overall patients (Table 2).

**Table 1:** Patient baseline demographics and clinical characteristics

Characteristics	Overall N = 444	EM n = 261	CM n = 183
Age (years), mean (SD)	45.89 (11.81)	46.34 (11.35)	45.23 (12.44)
Gender (women), n (%)	381 (85.8%)	222 (85.1%)	159 (86.9%)
Years since migraine diagnosis, mean (SD)	23.34 (13.58)	22.52 (12.92)	24.52 (14.43)
Number of monthly migraine headache days, mean (SD)	13.12 (5.90)	9.30 (2.84)	18.58 (4.72)
WPAI absenteeism score*, mean (SD)	9.37 (17.85)	8.22 (15.65)	11.03 (20.57)
WPAI presenteeism score*, mean (SD)	42.75 (23.24)	38.70 (24.65)	48.70 (19.62)
WPAI overall work productivity loss score*, mean (SD)	46.86 (25.69)	42.42 (26.92)	53.29 (22.39)
WPAI non-work-related activity impairment score, mean (SD)	50.97 (23.91)	48.01 (24.96)	55.19 (21.71)
MSQ-RFR score, mean (SD)	45.05 (17.16)	48.05 (15.79)	40.77 (18.15)
MSQ-RFP score, mean (SD)	63.78 (19.15)	65.38 (18.78)	61.50 (19.49)

*Scores were computed only for employed individuals

Mean (SD) was calculated based on non-missing values. WPAI scores are presented as % as units

EM was defined as 4 to 14 migraine headache days and < 15 headache days per 30-day period in the prospective baseline period. In the event that there were any patients with 4 to < 8 migraine headache days and 15–29 headache days per 30-day period in the prospective baseline period, they were considered to have episodic migraine

CM was defined as 15–29 headache days per 30-day period in the prospective baseline period, of which at least 8 are migraine

*CM* chronic migraine, *EM* episodic migraine, *MSQ* Migraine-Specific Quality of Life questionnaire, *N* total number of patients, *PRO* patient-reported outcome, *RFP* role function-preventive, *RFR* role function-restrictive, *SD* standard deviation, *WPAI* Work Productivity and Activity Impairment questionnaire

### Reliability

Test–retest reliability results, estimated in stable patients treated with placebo, showed moderate correlations for WPAI presenteeism (ICC: all patients = 0.438; EM patients = 0.227; CM patients = 0.893) and WPAI non-work-related Activity Impairment (ICC: all patients = 0.446; EM patients = 0.272; CM patients = 0.653) and a fair correlation for WPAI overall work productivity loss (ICC: all patients = 0.360; EM patients = 0.158; CM patients = 0.885) [42].

### Validity

At baseline, all the correlations between WPAI domain scores and continuous anchor variables exceeded the recommended threshold of ≥ 0.30, except for WPAI domain scores with the number of monthly migraine headache days in the overall population, and in patients with EM or CM. In patients with EM, the correlation between WPAI non-work-related activity impairment and MSQ-RFR was moderate-to-high (−0.606, Additional file 2: Figure S1).

### Responsiveness

WPAI domain scores improved (decreased) from baseline to Month 3 (Additional file 2: Table S1). Overall, patients achieving the pre-specified responsiveness thresholds for the monthly migraine headache days, and MSQ-RFP, MSQ-RFR from baseline to Month 3 (responders) showed significant improvements in WPAI domain scores compared with non-responders (*P* < 0.001, Table 3). Similar results were reported in patients with EM and CM (non-responders vs non-responders, *P* < 0.05) (Additional file 2: Table S2A and Table S2B).

**Table 2:** Patient baseline demographics and clinical characteristics in the employed patients

Characteristics	Overall N = 289	EM n = 171	CM n = 118
Age (years), mean (SD)	43.60 (10.04)	43.82 (10.00)	43.28 (10.15)
Gender (women), n (%)	245 (84.8)	143 (83.6)	102 (86.4)
Years since migraine diagnosis, mean (SD)	22.11 (12.45)	21.72 (12.55)	22.67 (12.33)
Number of monthly migraine headache days, mean (SD)	13.17 (5.73)	9.57 (2.82)	18.40 (4.78)
WPAI absenteeism score, mean (SD)	9.37 (17.85)	8.22 (15.65)	11.03 (20.57)
WPAI presenteeism score, mean (SD)	42.75 (23.24)	38.70 (24.65)	48.70 (19.62)
WPAI overall work impairment score, mean (SD)	46.86 (25.69)	42.42 (26.92)	53.29 (22.39)
WPAI non-work-related activity impairment score, mean (SD)	49.24 (23.22)	46.90 (24.31)	52.63 (21.18)
MSQ-RFR score, mean (SD)	46.21 (16.01)	48.29 (14.82)	43.20 (17.22)
MSQ-RFP score, mean (SD)	66.51 (16.93)	67.11 (17.27)	65.64 (16.47)

Mean (SD) was calculated based on non-missing values. WPAI scores are presented as % as units

EM was defined as 4 to 14 migraine headache days and < 15 headache days per 30-day period in the prospective baseline period. In the event that there were any patients with 4 to < 8 migraine headache days and 15–29 headache days per 30-day period in the prospective baseline period, they were considered to have episodic migraine

CM was defined as 15–29 headache days per 30-day period in the prospective baseline period, of which at least 8 are migraine

*CM* chronic migraine, *EM* episodic migraine, *MSQ* Migraine-Specific Quality of Life questionnaire, *N* total number of patients, *PRO* patient-reported outcome, *RFP* role function-preventive, *RFR* role function-restrictive, *SD* standard deviation, *WPAI* Work Productivity and Activity Impairment questionnaire

### Meaningful within-patient change thresholds

The AUC for change in WPAI domain scores ranged from 0.7 to 0.8 for all the selected anchor groups, indicating “acceptable” ability of the model to differentiate between responder and non-responder groups (Additional file 2: Table S4).

**Fig. 1:**
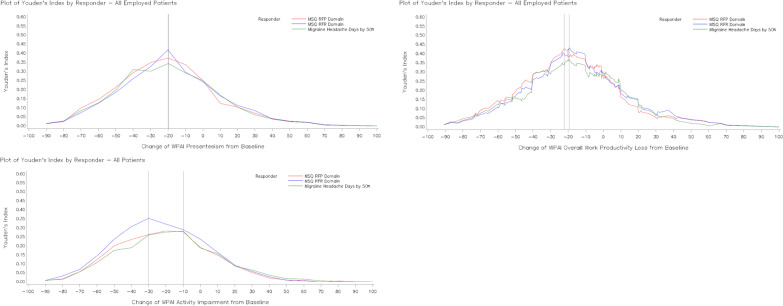
Youden’s index for WPAI domain scores improvement at Month 3 for selected anchors (Overall population). WPAI scores are presented as % unit. Abbreviations: *MHD* monthly migraine headache days, *MSQ* Migraine-Specific Quality of Life questionnaire, *RFP* Role Function-Preventive, *RFR* Role Function-Restrictive, *WPAI* Work Productivity and Activity Impairment questionnaire, *YI* Youden index

**Fig. 2:**
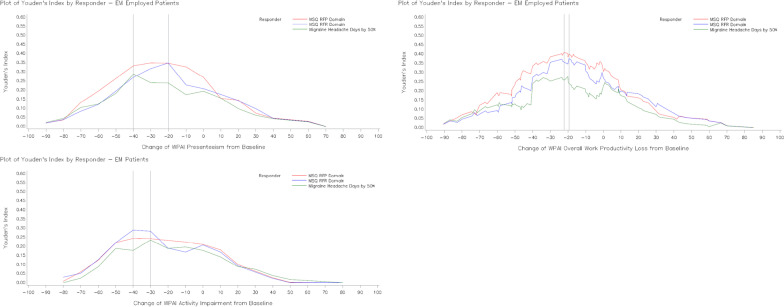
Youden’s index for WPAI domain scores improvement at Month 3 for selected anchors (EM). WPAI scores are presented as % unit. Abbreviations: *EM* episodic migraine, *MHD* monthly migraine headache days, *MSQ* Migraine-Specific Quality of Life questionnaire, *RFP* Role Function-Preventive, *RFR* Role Function-Restrictive, *WPAI* Work Productivity and Activity Impairment questionnaire, *YI* Youden index

**Fig. 3:**
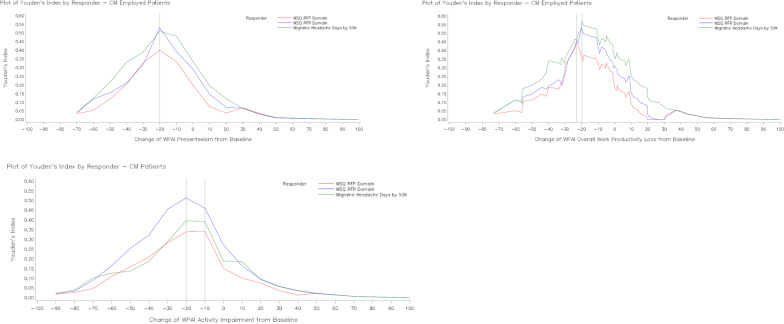
Youden’s index for WPAI domain scores improvement at Month 3 for selected anchors (CM). WPAI scores are presented as % unit. *CM* chronic migraine, *MHD* monthly migraine headache days, *MSQ* Migraine-Specific Quality of Life questionnaire, *RFP* role function-preventive, *RFR* role function-restrictive, *WPAI* Work Productivity and Activity Impairment questionnaire, *YI* Youden index

In the overall population, an optimum meaningful change in WPAI domain scores (% unit) from baseline to Month 3 corresponding to maximum YI of all three anchors was −20.0 for presenteeism, −22.2 to −19.4 for overall work productivity loss and −30.0 to −10.0 for non-work-related activity impairment (Fig. 1). In patients with EM, optimum meaningful change was −40.0 to −20.0 for presenteeism and −22.2 to −19.3 for overall work productivity loss and −40.0 to −30.0 for non-work-related activity impairment (Fig. 2). In patients with CM, optimum meaningful change was −20.0 for presenteeism and −23.0 to −19.8 for overall work productivity loss and −20.0 to −10.0 for non-work-related activity impairment (Fig. 3).

For the overall population, Phi coefficient was ≥ 0.24 and sensitivity ranged from 0.4 to 0.8 (Table 4). YI was the highest for cut-off points of approximately -20 unit-change from baseline in WPAI (% unit) for the anchor groups, Table 3. In patients with EM, a similar trend was followed only for WPAI presenteeism and WPAI overall work productivity loss domains (Additional file 2: Table S3A). In patients with CM, results for YI were comparable to that for overall population (Additional file 2: Table S3B). The meaningful change threshold of −20 (% unit) was chosen based on the overlapping data on Youden index, Phi coefficient and sensitivity.

**Table 3:** Change in WPAI from baseline to Month 3 for responders and non-responders (Overall population)

WPAI	Variables	Responder/non-responder	N (%)	LSM change in WPAI domain scores from baseline to month 3			LSM change difference in WPAI domain scores between non-responder and responder	
				LSM change (SE)	Within group *P*-value		LSM change difference (SE)	*P*-value
WPAI presenteeism	MSQ-RFP domain	Non-responder	197 (69.4)	−1.211 (1.591)	0.4472		−	−
		Responder	87 (30.6)	−24.269 (2.396)	< 0.0001		23.058 (2.878)	0.0000
	MSQ-RFR domain	Non-responder	204 (71.8)	−1.742 (1.569)	0.2678		−	−
		Responder	80 (28.2)	−24.933 (2.506)	< 0.0001		23.191 (2.957)	0.0000
	Monthly migraine headache days by 50%	Non-responder	213 (75.0)	−2.450 (1.546)	0.1142		−	−
		Responder	71 (25.0)	−25.750 (2.678)	< 0.0001		23.300 (3.092)	0.0000
WPAI overall work productivity loss	MSQ-RFP domain	Non-responder	200 (69.2)	−1.872 (1.715)	0.2760		−	−
		Responder	89 (30.8)	−25.459 (2.574)	< 0.0001		23.587 (3.096)	0.0000
	MSQ-RFR domain	Non-responder	207 (71.6)	−2.246 (1.683)	0.1830		−	−
		Responder	82 (28.4)	−26.529 (2.674)	< 0.0001		24.283 (3.160)	0.0000
	Monthly migraine headache days by 50%	Non-responder	215 (74.4)	−3.343 (1.682)	0.0479		−	−
		Responder	74 (25.6)	−25.968 (2.868)	< 0.0001		22.626 (3.326)	0.0000
WPAI non-work-related activity impairment	MSQ-RFP domain	Non-responder	293 (66.0)	−7.470 (1.445)	< 0.0001		−	−
		Responder	151 (34.0)	−24.710 (2.015)	< 0.0001		17.239 (2.484)	0.0000
	MSQ-RFR Domain	Non-responder	309 (69.6)	−6.353 (1.352)	< 0.0001		−	−
		Responder	135 (30.4)	−29.310 (2.045)	< 0.0001		22.957 (2.452)	0.0000
	Monthly migraine headache days by 50%	Non-responder	319 (71.8)	−7.616 (1.364)	< 0.0001		−	−
		Responder	125 (28.2)	−27.923 (2.179)	< 0.0001		20.307 (2.571)	0.0000

ANCOVA models were utilized to examine differences in the change in WPAI presenteeism, overall productivity loss and non-work-related activity impairment from Baseline to Month 3 among patients in all anchor-based responder groups

ANCOVA model: change in WPAI = anchor responder + baseline WPAI

WPAI scores are presented as % unit

*ANCOVA* analysis of covariance, *LSM* least squares mean, *MSQ* Migraine-Specific Quality of Life questionnaire, *RFP* role function-preventive, *RFR* role function-restrictive, *SE* standard error, *WPAI* Work Productivity and Activity Impairment questionnaire

**Table 4:** ROC analyses for change in WPAI domain scores from baseline to Month 3 (Overall population)

WPAI	Dependent variable	Cut-off point	Sensitivity	Specificity	Positive predictive value	Negative predictive value	Youden index	Phi coefficient
WPAI presenteeism	Monthly migraine headache days by 50%	−39.96	0.4085	0.9014	0.5800	0.8205	0.3099	0.3523
		−29.97	0.4789	0.8216	0.4722	0.8255	0.3005	0.2991
		−19.98	0.6197	0.7230	0.4272	0.8508	0.3427	0.3087
	MSQ-RFP domain	−30.00	0.4943	0.8528	0.5972	0.7925	0.3470	0.3677
		−20.00	0.6207	0.7513	0.5243	0.8177	0.3720	0.3566
		−10.00	0.7471	0.5888	0.4452	0.8406	0.3360	0.3099
	MSQ-RFR domain	−29.99	0.4875	0.8382	0.5417	0.8066	0.3257	0.3368
		−19.99	0.6625	0.7549	0.5146	0.8508	0.4174	0.3905
		−10.00	0.7250	0.5686	0.3973	0.8406	0.2936	0.2643
WPAI overall work productivity loss	Monthly migraine headache days by 50%	−20.09	0.5541	0.8093	0.5000	0.8406	0.3634	0.3518
		−19.79	0.6216	0.7488	0.4600	0.8519	0.3705	0.3399
		−19.37	0.6216	0.7442	0.4554	0.8511	0.3658	0.3348
	MSQ-RFP domain	−22.17	0.5730	0.8550	0.6375	0.8182	0.4280	0.4416
		−22.02	0.5730	0.8500	0.6296	0.8173	0.4230	0.4348
		−20.12	0.5730	0.8450	0.6220	0.8164	0.4180	0.4281
	MSQ-RFR domain	−20.01	0.6463	0.7778	0.5354	0.8474	0.4241	0.4029
		−19.39	0.6585	0.7729	0.5347	0.8511	0.4315	0.4080
		−19.16	0.6585	0.7681	0.5294	0.8503	0.4267	0.4025
WPAI activity impairment	Monthly migraine headache days by 50%	−29.95	0.5040	0.7555	0.4468	0.7954	0.2595	0.2507
		−19.97	0.6480	0.6270	0.4050	0.8197	0.2750	0.2485
		−9.98	0.8000	0.4796	0.3759	0.8596	0.2796	0.2566
	MSQ-RFP domain	−29.98	0.4901	0.7713	0.5248	0.7459	0.2614	0.2660
		−19.99	0.6358	0.6451	0.4800	0.7746	0.2808	0.2674
		−10.00	0.7815	0.4949	0.4436	0.8146	0.2763	0.2671
	MSQRFR domain	−40.01	0.4296	0.8770	0.6042	0.7787	0.3067	0.3427
		−30.01	0.5630	0.7896	0.5390	0.8053	0.3526	0.3484
		−20.00	0.6741	0.6472	0.4550	0.8197	0.3213	0.2971

Logistic regression was performed with selected anchor as a dependent variable and each WPAI score as an independent variable. Sensitivity, specificity, positive predictive value, negative predictive value, and Phi correlation for change scores above and below the optimal cut-point were provided to show the range of alternative WPAI change score thresholds. WPAI scores are presented as % unit

*MSQ* Migraine-Specific Quality of Life questionnaire, *RFP* role function-preventive, *RFR* role function-restrictive, *ROC* receiver-operator characteristic, *WPAI* Work Productivity and Activity Impairment questionnaire

The eCDF plots showed a clear separation between the curves representing anchor responder and non-responder groups by WPAI change scores (Additional file 2: Figure S2).

Using the established meaningful change threshold of −20 (% unit) for WPAI, the percentage of patients from the non-responder anchor group (no change/worsening) that were mis-classified as experiencing meaningful improvement in WPAI were between 22.2 and 37.3% for overall population, 25.7% to 44.7% for EM and 16.3% to 27.9% for CM, respectively.

## Discussion

This post-hoc analyses of the CONQUER study demonstrated the reliability, validity, responsiveness, and meaningful change of the WPAI questionnaire in patients with EM or CM. These results support the validity of the WPAI as a PRO to measure the impact of EM or CM on work productivity (overall and presenteeism) and non-work-related activity impairment. The meaningful change thresholds for the WPAI domain scores may help in evaluating clinically meaningful treatment responses and may be appropriate for inclusion of WPAI in future preventive migraine clinical studies.

Test–retest reliability results showed moderate correlations for WPAI presenteeism and WPAI non-work-related activity impairment and a fair correlation for WPAI overall work productivity loss. The modest ICCs for the WPAI may reflect the relatively brief, one-week recall interval. As the number of migraine days and their severity is known to vary within person from time to time, the instability of the phenomenon being measured may contribute to modest ICCs. This may also account for the higher ICCs we observed for CM than for EM. The greater ICCs among patients with CM than patients with EM may be due to the small sample size or the shorter recall period. A longer recall period longer for patients with EM may provide a more stable measurement of reliability.

Construct validity was confirmed between WPAI domain scores and continuous anchor variables- monthly migraine headache days, MSQ-RFR, and MSQ-RFP. Using an anchor-based method, a threshold of -20-unit change in scores at Month 3 (from baseline) was found to be meaningful for the three WPAI domain scores. The identified responder thresholds were consistent across patients with EM or CM. The eCDF plots substantiated the findings of the selected meaningful change threshold. A meaningful within-patient responder threshold was defined for three of the WPAI domains (presenteeism, overall work productivity, and non-work-related activity impairment) for patients with migraine (EM or CM).

The reliability, validity, responsiveness, and meaningful change of the WPAI among patients with migraine had not been previously established. The findings of this research indicate that this instrument is appropriate for use in this population and provides a within-patient responder threshold that can be used to determine a meaningful change in presenteeism, overall work productivity and non-work-related activity impairment. This threshold can be used in future research to determine the proportion of patients that are experiencing a degree of improvement that is impactful for work and non-work activities.

In the present analyses, WPAI absenteeism sub scores alone (i.e. without accounting for presenteeism) were not considered due to a possible lack of sensitivity in patients with migraine. This may be explained by a low mean baseline score of 9.37 and smaller change from baseline of −2.83 for absenteeism versus other domains making the results less sensitive to the present analyses. These low mean absenteeism scores are not unexpected, as qualitative research has revealed that employed patients with migraine frequently ‘power-through’ work, which results in modest levels of absenteeism. When people miss enough work, they may become unemployed. Across a range of chronic pain disorders unemployment rates increase with pain severity and in the highest grades of pain unemployment rates reach 20%. Selecting for persons who are employed may in effect select against individuals with high rates of absenteeism. Presenteeism is strongly influenced by migraine [28, 33, 45]. Similar findings have been observed in a prior study, where patients with migraine were more likely to miss non-work activities (family, social, and leisure) than work [46]. Prior work has addressed the psychometric properties of the WPAI for other disease states using similar methods to the those used in the present report. This research also used anchor-based and distribution-based methods and utilized clinical trial data. However, unlike the current research, the previous research determined *apriori* to not estimate thresholds for absenteeism and presenteeism separately, classifying these as individual domains that should be used for descriptive purposes only [47]. Another study also used clinical trial data to evaluate the change in all four WPAI domain scores among patients with ankylosing spondylitis. However, the research evaluated WPAI responsiveness using minimum clinically important differences from two other PROs, which were quantified with standardized response mean (SRM) calculations [48]. Although, all WPAI domain scores were evaluated in patients with ankylosing spondylitis, absenteeism domains was observed to be less sensitive for identifying meaningful changes [48], similar to the present study.

Strengths of this study include the use of current methods for evaluating psychometric properties, determining meaningful within-patient change thresholds, and the PROs for the analyses were comprehensive including a quality-of-life measure and an electronic daily diary measure of MHDs.

There are certain limitations to our study. One of the limitations is that the patient population for the CONQUER clinical trial was specific to patients with migraine who had a history of two to four preventive treatment category failures. In addition, analyses were limited to people with ≥ 4 monthly migraine headache days, which may represent a more impacted sample than the general population. This higher rate of monthly migraine days may have influenced employment status and associated disability, and therefore results may not be generalizable to a sample with fewer monthly migraine days. Moderate reliability or greater was not observed for the WPAI in this patient population, perhaps because the number of headache days varies from week to week and the recall interval for the WPAI is only one week. This study included WPAI data at baseline and Month 3, hence we could not include Month 1 data for analysis. Also, there was no information from the patient perspective regarding what constitutes an important change in WPAI scores. The analysis plan evaluated reliability in a group with no change or a change of only one day over one month when the WPAI evaluated one week of that month; real within person change may have reduced the ICCs for the WPAI. Responsiveness for WPAI absenteeism was not evaluated because of very low score in the patient population. Additionally, the MSQ-RFR and MSQ-RFP as anchors used in the study to validate WPAI should be easy to interpret. Change from baseline is monthly migraine days is a widely used, included in labelling, and recommended in guidelines as a primary endpoint in preventive treatment trials [9, 38]. MSQ-RFP is a recommended secondary endpoint and is included in labelling for migraine medications [49, 50]. In addition to the lack of temporal alignment to the MHD measure used to define no change and the WPAI, the one-week recall interval itself may have had reduced ICCs because of actual variation from week to week in presenteeism and non-work-related activity impairment. Increasing the recall interval reduces temporal sampling error but may result in recall bias. Reilly et al. reported that WPAI score may be unduly influenced by the most severe disease day in that interval and suggested that use of recall period as short as 24 h may be warranted to reduce recall bias [14]. Daily measures over longer periods mitigate both temporal sampling error and recall bias but substantially increase participant burden and may induce fatigue. For a disorder such as migraine, characterized by attacks, reliability may be improved with longer sampling intervals or by averaging multiple 7-day sampling intervals.

There is uncertainty if these results can be generalized to the overall migraine population. However, results observed in patients with EM or CM were consistent. Therefore, the identified responder thresholds can be applied to migraine populations without preventive treatment failures until further research is completed to confirm (or contradict) this recommendation. Prior publications show that patients with high frequency EM (10–14 headache days/month) have similar disability to those of CM (≥ 15 headache days/month) [51, 52]. On the other hand, Ishii et al., in their study comparing chronic versus episodic migraine concluded that patients with chronic migraine (15–23 headache days/month) have significantly greater disability compared to patients with high-frequency episodic migraine (8–14 headache days/month) (median [interquartile range] MIDAS score: 55 [30–90] vs. 38 [20–58], *p* < 0.001) [53]. Considering patients with low frequency EM were not included in the current analysis, it is not known if responder data of patients with low frequency EM would be comparable to those with low frequency CM or not. Future efforts may compare the meaningful change thresholds from this study with other studies with a less restrictive migraine population, and an evaluation of the meaningfulness of the change via patient input would be informative. Previous studies have used 50% threshold for monthly migraine headache days in patients with CM [12], which was confirmed by in post-hoc analysis of a preventive anti-CGRP treatment for CM [54]. Data on the proportion of patients achieving the responder threshold for galcanezumab versus placebo may be considered in a future publication.

## Conclusions

The results of this study demonstrated the validity of the WPAI PRO to measure the impact of EM or CM on work productivity (overall and presenteeism) and non-work-related activity impairment. The meaningful change thresholds for the WPAI domain scores may help in evaluating clinically meaningful treatment responses and may be appropriate for inclusion of WPAI in future preventive migraine clinical studies. The analysis was specific to a population with a history of multiple standard-of-care migraine preventive medication failures and moderate frequency EM or greater; however, the meaningful within-patient threshold may be applied to migraine research studies until generalizability of these findings are confirmed.

## Supplementary Information

**Additional file 1**. WPAI Questions and Score.

**Additional file 2. Table S1**. Change from baseline to Month 3 in WPAI domain scores. **Table S2.** Change in WPAI domain scores from baseline to Month 3 for responders and non-responders (EM). **Table S3**. Change in WPAI domain scores from baseline to Month 3 for responders and non-responders (CM). **Table S4**. ROC analyses for change in WPAI domain scores from baseline to Month 3 (EM). **Table S5**. ROC analyses for change in WPAI domain scores from baseline to Month 3 (CM). **Table S6**. Logistic regression of improvement in anchors on change from baseline to month 3 in WPAI domain scores. **Figure S1**. Spearman correlations of baseline WPAI domain scores with MSQ, and monthly migraine headache days. **A** Correlation of WPAI Scores with MSQ_RFP, MSQ_RFR, and Migraine Headache Days—All Patients. **B** Correlation of WPAI Scores with MSQ_RFP, MSQ_RFR, and Migraine Headache Days—EM Patients. **C** Correlation of WPAI Scores with MSQ_RFP, MSQ_RFR, and Migraine Headache Days—CM Patients. **Figure S2**. Cumulative distribution function plots for change in WPAI from Baseline to Month 3. **A** WPAI presenteeism and improvement of MSQ-RFP domain—Overall. **B** WPAI presenteeism and improvement of MSQ-RFP domain—EM. **C** WPAI presenteeism and improvement of MSQ-RFP domain—CM. **D** WPAI presenteeism and improvement of MSQ-RFR domain—Overall. **E** WPAI presenteeism and improvement of MSQ-RFR domain—EM. **F** WPAI presenteeism and improvement of MSQ-RFR domain—CM. **G** WPAI presenteeism and improvement of monthly headache days 50%—Overall. **H** WPAI presenteeism and improvement of monthly headache days 50%—EM. **I** WPAI presenteeism and improvement of monthly headache days 50%—CM. **J** WPAI overall work productivity loss and improvement of migraine headache days 50%—Overall. **K** WPAI overall work productivity loss and improvement of migraine headache days 50%—EM. **L** WPAI overall work productivity loss and improvement of migraine headache days 50%—CM. **M** WPAI overall work productivity loss and improvement of MSQ-RFR—Overall. **N** WPAI overall work productivity loss and improvement of MSQ-RFR—EM. **O** WPAI overall work productivity loss and improvement of MSQ-RFR—CM. **P** WPAI overall work productivity loss and improvement of MSQ-RFP—Overall. **Q** WPAI overall work productivity loss and improvement of MSQ-RFP—CM. **R** WPAI non-work-related activity impairment and improvement of migraine headache days-50%—Overall. **S** WPAI non-work-related activity impairment and improvement of migraine headache days-50%—EM. **T** WPAI non-work-related activity impairment and improvement of migraine headache days-50%—CM.

## Data Availability

Eli Lilly and Company provides access to all individual participant data collected during the trial, after anonymization except for pharmacokinetic or genetic data. Data are available to request six months after the indication studied has been approved in the United States and European Union and after primary publication acceptance, whichever is later. No expiration date of data requests is currently set once data are made available. Access is provided after a proposal has been approved by an independent review committee identified for this purpose and after receipt of a signed data sharing agreement. Data and documents, including the study protocol, statistical analysis plan, clinical study report, blank or annotated case report forms, will be provided in a secure data sharing environment. For details on submitting a request, see the instructions provided at www.vivli.org.

## Abbreviations

ANOVA: Analysis of variance
AUC: Area under the curve
CM: Chronic migraine
C: Concordance
eCDF: Empirical cumulative distribution function
EM: Episodic migraine
ICCs: Intra-class correlation coefficients
ICHD-3: International Classification of Headache Disorders
MSQ: Migraine-Specific Quality of Life questionnaire
N: Number of patients
n: Number of patients in a subgroup
WPAI_ova: Overall work productivity loss
PRO: Patient-reported outcome
ROC: Receiver-operator characteristic
RFP: Role function preventive
RFR: Role function restrictive
SD: Standard deviation
SE: Standard error
SRM: Standardized response means
WPAI: Work Productivity and Activity Impairment
WPAI_act: WPAI non-work-related activity impairment
WPAI_pres: WPAI presenteeism
YI: Youden index
