# Supplementary material for: Validation and meaningful within-patient change in work productivity and activity impairment questionnaire (WPAI) for episodic or chronic migraine
Source: J Patient Rep Outcomes. 2023 Apr 4;7:34. doi: 10.1186/s41687-023-00552-4 (PMC10073392; doi:10.1186/s41687-023-00552-4)
Supplement: Supplementary file 1 — Additional file 1. WPAI Questions and Score. [file 41687_2023_552_MOESM1_ESM.docx]

# WPAI Questions and Score

**Supplementary File 1**: Calculation of WPAI absenteeism, presenteeism, work productivity loss, and non-work-related activity impairment

WPAI domain scores consisted of the following 6 items:

Q1) employment status,

Q2) hours missed from work due to migraine,

Q3) hours missed from work for other reasons,

Q4) hours actually worked,

Q5) degree migraine affected productivity while working (0-10, 0–no effect, 10- completely prevent to work), and

Q6) degree migraine affected productivity in regular unpaid activities (0-10, 0–no effect, 10-completely prevent me from doing).

Four scores were calculated from the responses to these 6 items as impairment percentages, with higher numbers indicating greater impairment and less productivity, i.e., worse outcomes.

• Absenteeism: Percent work time missed due to migraine (%) = Q2/ (Q2 + Q4) *100 for those who were currently employed.

• Presenteeism: The percent impairment while working due to migraine (%) = Q5*100/10 for those who were currently employed and worked in the past seven days

• Overall work productivity loss due to migraine (%) = (Q2 / (Q2 + Q4) + (1 - Q2/ (Q2 + Q4)) × (Q5/10)) * 100

for those who were currently employed.

• Non-work-related activity impairment: The percent non-work-related activity impairment due to migraine (%) =Q6 * 100/10 for all respondents.
