# Supplementary material for: Validation and meaningful within-patient change in work productivity and activity impairment questionnaire (WPAI) for episodic or chronic migraine
Source: J Patient Rep Outcomes. 2023 Apr 4;7:34. doi: 10.1186/s41687-023-00552-4 (PMC10073392; doi:10.1186/s41687-023-00552-4)
Supplement: Supplementary file 2 — Additional file 2. Table S1. Change from baseline to Month 3 in WPAI domain scores. Table S2. Change in WPAI domain scores from baseline to Month 3 for responders and non-responders (EM). Table S3. Change in WPAI domain scores from baseline to Month 3 for responders and non-responders (CM). Table S4. ROC analyses for change in WPAI domain scores from baseline to Month 3 (EM). Table S5. ROC analyses for change in WPAI domain scores from baseline to Month 3 (CM). Table S6. Logistic regression of improvement in anchors on change from baseline to month 3 in WPAI domain scores. Figure S1. Spearman correlations of baseline WPAI domain scores with MSQ, and monthly migraine headache days. A Correlation of WPAI Scores with MSQ_RFP, MSQ_RFR, and Migraine Headache Days—All Patients. B Correlation of WPAI Scores with MSQ_RFP, MSQ_RFR, and Migraine Headache Days—EM Patients. C Correlation of WPAI Scores with MSQ_RFP, MSQ_RFR, and Migraine Headache Days—CM Patients. Figure S2. Cumulative distribution function plots for change in WPAI from Baseline to Month 3. A WPAI presenteeism and improvement of MSQ-RFP domain—Overall. B WPAI presenteeism and improvement of MSQ-RFP domain—EM. C WPAI presenteeism and improvement of MSQ-RFP domain—CM. D WPAI presenteeism and improvement of MSQ-RFR domain—Overall. E WPAI presenteeism and improvement of MSQ-RFR domain—EM. F WPAI presenteeism and improvement of MSQ-RFR domain—CM. G WPAI presenteeism and improvement of monthly headache days 50%—Overall. H WPAI presenteeism and improvement of monthly headache days 50%—EM. I WPAI presenteeism and improvement of monthly headache days 50%—CM. J WPAI overall work productivity loss and improvement of migraine headache days 50%—Overall. K WPAI overall work productivity loss and improvement of migraine headache days 50%—EM. L WPAI overall work productivity loss and improvement of migraine headache days 50%—CM. M WPAI overall work productivity loss and improvement of MSQ-RFR—Overall. N WPAI overall work produ [file 41687_2023_552_MOESM2_ESM.docx]

**Supplementary Table 1.** Change from baseline to Month 3 in WPAI domain scores

| **WPAI domain** |  | **All patients** | **Patients with EM** | **Patients with CM** |
| --- | --- | --- | --- | --- |
| *WPAI absenteeism* | N | 315 | 188 | 127 |
|  | Mean (SD) | -2.83 (16.69) | -3.59 (17.97) | -1.73 (14.64) |
|  | Missing, n (%) | 26 (8.25) | 17 (9.04) | 9 (7.09) |
|  | Ceiling, n (%) | 0 (0) | 0 (0) | 0 (0) |
| *WPAI presenteeism* | N | 315 | 188 | 127 |
|  | Mean (SD) | -8.27 (28.64) | -10.83 (30.27) | -4.52 (25.73) |
|  | Missing, n (%) | 31 (9.84) | 19 (10.11) | 12 (9.45) |
|  | Ceiling, n (%) | 1 (0.32) | − | 1 (0.79) |
| *WPAI overall work productivity loss* | N | 315 | 188 | 127 |
|  | Mean (SD) | -9.14 (30.97) | -11.88 (33.66) | -5.16 (26.21) |
|  | Missing, n (%) | 26 (8.25) | 17 (9.04) | 9 (7.09) |
|  | Ceiling, n (%) | 1 (0.32) | − | 1 (0.79) |
| *WPAI non-work-related activity impairment* | N | 463 | 270 | 193 |
|  | Mean (SD) | -13.33 (29.70) | -16.17 (30.42) | -9.29 (28.23) |
|  | Missing, n (%) | 19 (4.10) | 9 (3.33) | 10 (5.18) |
|  | Ceiling, n (%) | 1 (0.22) | − | 1 (0.52) |

WPAI scores are presented as % unit. “-” indicates missing data. Abbreviations: CM, chronic migraine; EM, episodic migraine; N, number of patients; n, number of patients in a subgroup, SD, standard deviation; WPAI, Work Productivity and Activity Impairment questionnaire.

**Supplementary Table 2.** Change in WPAI domain scores from baseline to Month 3 for responders and non-responders (EM)

|  | | | **LSM change in WPAI scores from baseline** | | **LS mean change difference in WPAI scores between non-responder and responder** | |
| --- | --- | --- | --- | --- | --- | --- |
| **WPAI domain** | **Variables** | **Responder/**  **non-responder** | **LSM change (SE)** | **Within group**  **P-value** | **LS mean change**  **difference (SE)** | **P-value** |
| *WPAI presenteeism* | MSQ-RFP domain | Non-responder | -3.057 (2.044) | 0.1366 | − | − |
|  |  | Responder | -26.099 (2.871) | <0.0001 | 23.042 (3.536) | 0.0000 |
|  | MSQ-RFR domain | Non-responder | -4.438 (2.096) | 0.0357 | − | − |
|  |  | Responder | -24.075 (3.023) | <0.0001 | 19.637 (3.686) | 0.0000 |
|  | Monthly migraine headache days by 50% | Non-responder | -5.495 (2.044) | 0.0079 | − | − |
|  |  | Responder | -24.672 (3.293) | <0.0001 | 19.177 (3.876) | 0.0000 |
| *WPAI overall work productivity loss* | MSQ-RFP domain | Non-responder | -3.570 (2.225) | 0.1105 | − | − |
|  |  | Responder | -28.077 (3.114) | <0.0001 | 24.507 (3.842) | 0.0000 |
|  | MSQ-RFR Domain | Non-responder | -4.882 (2.269) | 0.0329 | − | − |
|  |  | Responder | −26.256 (3.259) | <0.0001 | 21.374 (3.982) | 0.0000 |
|  | Monthly migraine headache days by 50% | Non-responder | -6.596 (2.242) | 0.0037 | − | − |
|  |  | Responder | -25.427 (3.588) | <0.0001 | 18.831 (4.231) | 0.0000 |
| *WPAI non-work-related activity impairment* | MSQ-RFP Domain | Non-responder | -10.359 (1.926) | <0.0001 | − | − |
|  |  | Responder | -26.320 (2.549) | <0.0001 | 15.962 (3.202) | 0.0000 |
|  | MSQ-RFR Domain | Non-responder | -9.823 (1.843) | <0.0001 | − | − |
|  |  | Responder | -28.859 (2.607) | <0.0001 | 19.036 (3.192) | 0.0000 |
|  | Monthly migraine headache days by 50% | Non-responder | -10.578 (1.837) | <0.0001 | − | − |
|  |  | Responder | -28.372 (2.715) | <0.0001 | 17.794 (3.279) | 0.0000 |

WPAI scores are presented as % unit.

ANCOVA models were utilized to examine differences in the change in WPAI presenteeism, overall productivity loss and non-work-related activity impairment from Baseline to Month 3 among patients in all anchor-based responder groups. The change in WPAI presenteeism, overall productivity loss, and non-work-related activity impairment were used as the dependent variable, while the responder group variable was used as the independent variable. Baseline WPAI presenteeism, overall productivity loss, and non-work-related activity impairment were adjusted for in the respective models. This was done by using SAS PROC MIXED.

Abbreviations: ANCOVA, analysis of covariance; EM, episodic migraine; LSM, least square mean; MSQ, Migraine-Specific Quality of Life questionnaire; RFP, Role Function-Preventive; RFR, Role Function-Restrictive; SE, standard error; WPAI, Work Productivity and Activity Impairment questionnaire.

**Supplementary Table 3.** Change in WPAI domain scores from baseline to Month 3 for responders and non-responders (CM)

|  | | | **LS mean change in WPAI scores from baseline** | | | **LS mean change difference in WPAI scores between non-responder and responder** | |
| --- | --- | --- | --- | --- | --- | --- | --- |
| **WPAI domain** | **Variables** | **Responder/**  **non-responder** | **LSM change (SE)** | **Within group**  **P-value** | **LSM change**  **difference (SE)** | | **P-value** |
| *WPAI presenteeism* | MSQ-RFP domain | Non-responder | 0.594 (2.378) | 0.8032 | − | | − |
|  |  | Responder | -19.016 (4.002) | <0.0001 | 19.610 (4.656) | | 0.0001 |
|  | MSQ-RFR domain | Non-responder | 0.949 (2.227) | 0.6709 | − | | − |
|  |  | Responder | -24.216 (4.226) | <0.0001 | 25.164 (4.778) | | 0.0000 |
|  | Monthly migraine headache days by 50% | Non-responder | 1.302 (2.161) | 0.5480 | − | | − |
|  |  | Responder | -26.605 (4.211) | <0.0001 | 27.908 (4.735) | | 0.0000 |
| *WPAI overall work productivity loss* | MSQ-RFP domain | Non-responder | -0.493 (2.471) | 0.8422 | − | | − |
|  |  | Responder | -18.245 (4.140) | <0.0001 | 17.752 (4.822) | | 0.0004 |
|  | MSQ-RFR domain | Non-responder | 0.092 (2.318) | 0.9683 | − | | − |
|  |  | Responder | -23.730 (4.361) | <0.0001 | 23.822 (4.940) | | 0.0000 |
|  | Monthly migraine headache days by 50% | Non-responder | 0.943 (2.241) | 0.6747 | − | | − |
|  |  | Responder | -26.739 (4.230) | <0.0001 | 27.682 (4.797) | | 0.0000 |
| *WPAI non-work-related activity impairment* | MSQ-RFP domain | Non-responder | -4.020 (2.092) | 0.0562 | − | | − |
|  |  | Responder | -21.240 (3.155) | <0.0001 | 17.219 (3.792) | | 0.0000 |
|  | MSQ-RFR domain | Non-responder | -2.094 (1.870) | 0.2641 | − | | − |
|  |  | Responder | -29.526 (3.142) | <0.0001 | 27.432 (3.662) | | 0.0000 |
|  | Monthly migraine headache days by 50% | Non-responder | -4.063 (1.941) | 0.0377 | − | | - |
|  |  | Responder | -26.307 (3.503) | <0.0001 | 22.244 (4.005) | | 0.0000 |

WPAI scores are presented as % unit.

Abbreviations: ANCOVA, analysis of covariance, CM, chronic migraine, LSM, least squares mean; MSQ, Migraine-Specific Quality of Life questionnaire; PGI-S, Patient Global Impression of Severity; RFP, role function-preventive; RFR, role function-restrictive; SE, standard error; WPAI, Work Productivity and Activity Impairment questionnaire.

**Supplementary Table 4.** ROC analyses for change in WPAI domain scores from baseline to Month 3 (EM)

| **WPAI domain** | **Dependent variable** | **Cut-off point** | **Sensitivity** | **Specificity** | **Positive predictive value** | **Negative predictive value** | **Youden  index** | **Phi coefficient** |
| --- | --- | --- | --- | --- | --- | --- | --- | --- |
| *WPAI presenteeism* | Monthly migraine headache days by 50% | -40.02 | 0.4255 | 0.8607 | 0.5405 | 0.7955 | 0.2862 | 0.3101 |
|  |  | -30.01 | 0.4681 | 0.7705 | 0.4400 | 0.7899 | 0.2386 | 0.2342 |
|  |  | -20.01 | 0.5745 | 0.6639 | 0.3971 | 0.8020 | 0.2384 | 0.2178 |
|  | MSQ-RFP domain | -40.04 | 0.4386 | 0.8929 | 0.6757 | 0.7576 | 0.3315 | 0.3789 |
|  |  | -30.03 | 0.5263 | 0.8214 | 0.6000 | 0.7731 | 0.3477 | 0.3602 |
|  |  | -20.02 | 0.6316 | 0.7143 | 0.5294 | 0.7921 | 0.3459 | 0.3335 |
|  | MSQ-RFR domain | -40.00 | 0.4000 | 0.8684 | 0.5946 | 0.7500 | 0.2684 | 0.3041 |
|  |  | -30.00 | 0.5091 | 0.8070 | 0.5600 | 0.7731 | 0.3161 | 0.3245 |
|  |  | -20.00 | 0.6364 | 0.7105 | 0.5147 | 0.8020 | 0.3469 | 0.3314 |
| *WPAI non-work-related activity impairment* | Monthly migraine headache days by 50% | -49.97 | 0.3049 | 0.8827 | 0.5435 | 0.7349 | 0.1876 | 0.2285 |
|  |  | -29.98 | 0.5122 | 0.7207 | 0.4565 | 0.7633 | 0.2329 | 0.2263 |
|  |  | -10.00 | 0.7927 | 0.4022 | 0.3779 | 0.8090 | 0.1949 | 0.1909 |
|  | MSQ-RFP domain | -40.01 | 0.4105 | 0.8313 | 0.5821 | 0.7113 | 0.2419 | 0.2664 |
|  |  | -30.01 | 0.5053 | 0.7349 | 0.5217 | 0.7219 | 0.2402 | 0.2419 |
|  |  | -20.00 | 0.6526 | 0.5783 | 0.4697 | 0.7442 | 0.2309 | 0.2223 |
|  | MSQ-RFR domain | -49.97 | 0.3218 | 0.8966 | 0.6087 | 0.7256 | 0.2184 | 0.2702 |
|  |  | -39.98 | 0.4483 | 0.8391 | 0.5821 | 0.7526 | 0.2874 | 0.3101 |
|  |  | -29.98 | 0.5402 | 0.7414 | 0.5109 | 0.7633 | 0.2816 | 0.2779 |
| *WPAI overall work productivity loss* | Monthly migraine headache days by 50% | -33.89 | 0.4583 | 0.8130 | 0.4889 | 0.7937 | 0.2713 | 0.2769 |
|  |  | -23.37 | 0.5000 | 0.7724 | 0.4615 | 0.7983 | 0.2724 | 0.2660 |
|  |  | -20.07 | 0.5208 | 0.7561 | 0.4545 | 0.8017 | 0.2769 | 0.2664 |
|  | MSQ-RFP domain | -23.44 | 0.5690 | 0.8319 | 0.6346 | 0.7899 | 0.4008 | 0.4125 |
|  |  | -22.17 | 0.5862 | 0.8230 | 0.6296 | 0.7949 | 0.4092 | 0.4168 |
|  |  | -20.13 | 0.5862 | 0.8142 | 0.6182 | 0.7931 | 0.4004 | 0.4058 |
|  | MSQ-RFR domain | -23.37 | 0.5536 | 0.8174 | 0.5962 | 0.7899 | 0.3710 | 0.3784 |
|  |  | -19.35 | 0.6429 | 0.7304 | 0.5373 | 0.8077 | 0.3733 | 0.3589 |
|  |  | -18.16 | 0.6429 | 0.7217 | 0.5294 | 0.8058 | 0.3646 | 0.3496 |

WPAI scores are presented as % unit.

Abbreviations: MSQ, Migraine-Specific Quality of Life questionnaire; PGI-S, Patient Global Impression of Severity; RFP, role function-preventive; RFR, role function-restrictive; ROC, receiver-operator characteristic; WPAI, Work Productivity and Activity Impairment questionnaire.

**Supplementary Table 5.** ROC analyses for change in WPAI domain scores from baseline to Month 3 (CM)

| **WPAI domain** | **Dependent variable** | **Cut-off point** | **Sensitivity** | **Specificity** | **Positive predictive value** | **Negative predictive value** | **Youden  index** | **Phi coefficient** |
| --- | --- | --- | --- | --- | --- | --- | --- | --- |
| *WPAI presenteeism* | Monthly migraine headache days by 50% | -29.99 | 0.5000 | 0.8901 | 0.5455 | 0.8710 | 0.3901 | 0.4031 |
|  |  | -19.99 | 0.7083 | 0.8022 | 0.4857 | 0.9125 | 0.5105 | 0.4509 |
|  |  | -10.00 | 0.8333 | 0.6484 | 0.3846 | 0.9365 | 0.4817 | 0.3933 |
|  | MSQ-RFP domain | -30.02 | 0.4333 | 0.8941 | 0.5909 | 0.8172 | 0.3275 | 0.3656 |
|  |  | -20.02 | 0.6000 | 0.8000 | 0.5143 | 0.8500 | 0.4000 | 0.3817 |
|  |  | -10.01 | 0.7000 | 0.6353 | 0.4038 | 0.8571 | 0.3353 | 0.2958 |
|  | MSQ-RFR domain | -29.98 | 0.4400 | 0.8778 | 0.5000 | 0.8495 | 0.3178 | 0.3332 |
|  |  | -19.99 | 0.7200 | 0.8111 | 0.5143 | 0.9125 | 0.5311 | 0.4761 |
|  |  | -9.99 | 0.7600 | 0.6333 | 0.3654 | 0.9048 | 0.3933 | 0.3260 |
| *WPAI non-work-related activity impairment* | Monthly migraine headache days by 50% | -30.02 | 0.4884 | 0.8000 | 0.4286 | 0.8358 | 0.2884 | 0.2761 |
|  |  | -20.01 | 0.6744 | 0.7214 | 0.4265 | 0.8783 | 0.3958 | 0.3473 |
|  |  | -10.01 | 0.8140 | 0.5786 | 0.3723 | 0.9101 | 0.3925 | 0.3330 |
|  | MSQ-RFP domain | -30.01 | 0.4643 | 0.8189 | 0.5306 | 0.7761 | 0.2832 | 0.2947 |
|  |  | -20.00 | 0.6071 | 0.7323 | 0.5000 | 0.8087 | 0.3394 | 0.3237 |
|  |  | -10.00 | 0.7500 | 0.5906 | 0.4468 | 0.8427 | 0.3406 | 0.3140 |
|  | MSQ-RFR domain | -30.01 | 0.6042 | 0.8519 | 0.5918 | 0.8582 | 0.4560 | 0.4530 |
|  |  | -20.01 | 0.7500 | 0.7630 | 0.5294 | 0.8957 | 0.5130 | 0.4669 |
|  |  | -10.00 | 0.8542 | 0.6074 | 0.4362 | 0.9213 | 0.4616 | 0.4062 |
| *WPAI overall work productivity loss* | Monthly migraine headache days by 50% | -19.79 | 0.7308 | 0.8370 | 0.5588 | 0.9167 | 0.5677 | 0.5196 |
|  |  | -19.14 | 0.7308 | 0.8261 | 0.5429 | 0.9157 | 0.5569 | 0.5053 |
|  |  | -18.57 | 0.7308 | 0.8152 | 0.5278 | 0.9146 | 0.5460 | 0.4915 |
|  | MSQ-RFP domain | -23.97 | 0.5161 | 0.8966 | 0.6400 | 0.8387 | 0.4127 | 0.4445 |
|  |  | -22.97 | 0.5484 | 0.8966 | 0.6538 | 0.8478 | 0.4449 | 0.4725 |
|  |  | -21.97 | 0.5484 | 0.8851 | 0.6296 | 0.8462 | 0.4334 | 0.4541 |
|  | MSQ-RFR domain | -20.01 | 0.6923 | 0.8370 | 0.5455 | 0.9059 | 0.5293 | 0.4887 |
|  |  | -19.80 | 0.6923 | 0.8261 | 0.5294 | 0.9048 | 0.5184 | 0.4744 |
|  |  | -19.15 | 0.6923 | 0.8152 | 0.5143 | 0.9036 | 0.5075 | 0.4605 |

WPAI scores are presented as % unit.

Abbreviations: CM, chronic migraine; MSQ, migraine-specific quality of life questionnaire; RFP, role function-preventive; RFR, role function-restrictive; WPAI, Work Productivity and Activity Impairment questionnaire.

**Supplementary Table 6.** Logistic regression of improvement in anchors on change from baseline to month 3 in WPAI domain scores

| **Independent variable** | **Dependent variable** | **N** | **C-statistic** | **OR (95% CI)** | **P-value** |
| --- | --- | --- | --- | --- | --- |
| **Overall** |  |  |  |  |  |
| *WPAI presenteeism* | MSQ-RFP domain | 284 | 0.742 | 0.964 (0.953, 0.975) | <0.0001 |
|  | MSQ-RFR domain | 284 | 0.737 | 0.966 (0.954, 0.977) | <0.0001 |
|  | Monthly migraine headache days by 50% | 284 | 0.729 | 0.967 (0.956, 0.978) | <0.0001 |
| *WPAI overall work productivity loss* | MSQ-RFP domain | 289 | 0.738 | 0.968 (0.958, 0.978) | <.0001 |
|  | MSQ-RFR domain | 289 | 0.740 | 0.969 (0.959, 0.979) | <0.0001 |
|  | Monthly migraine headache days by 50% | 289 | 0.725 | 0.972 (0.962, 0.982) | <0.0001 |
| *WPAI non-work-related activity impairment* | MSQ-RFP domain | 444 | 0.696 | 0.975 (0.968, 0.983) | <0.0001 |
|  | MSQ-RFR domain | 444 | 0.732 | 0.969 (0.961, 0.978) | <0.0001 |
|  | Monthly migraine headache days by 50% | 444 | 0.690 | 0.976 (0.968, 0.984) | <0.0001 |
| **Patients with EM** |  |  |  |  |  |
| *WPAI presenteeism* | MSQ-RFP domain | 169 | 0.751 | 0.963 (0.950, 0.977) | <0.0001 |
|  | MSQ-RFR domain | 169 | 0.711 | 0.971 (0.958, 0.984) | <0.0001 |
|  | Monthly migraine headache days by 50% | 169 | 0.675 | 0.976 (0.964, 0.989) | 0.0002 |
| *WPAI overall work productivity loss* | MSQ-RFP domain | 171 | 0.757 | 0.968 (0.956, 0.980) | <.0001 |
|  | MSQ-RFR domain | 171 | 0.722 | 0.973 (0.962, 0.985) | <0.0001 |
|  | Monthly migraine headache days by 50% | 171 | 0.665 | 0.982 (0.971, 0.993) | 0.0010 |
| *WPAI non-work-related activity impairment* | MSQ-RFP domain | 261 | 0.682 | 0.977 (0.968, 0.987) | <0.0001 |
|  | MSQ-RFR domain | 261 | 0.676 | 0.978 (0.968, 0.987) | <0.0001 |
|  | Monthly migraine headache days by 50% | 261 | 0.654 | 0.981 (0.972, 0.990) | <0.0001 |
| **Patients with CM** |  |  |  |  |  |
| *WPAI presenteeism* | MSQ-RFP domain | 115 | 0.715 | 0.965 (0.946, 0.985) | 0.0006 |
|  | MSQ-RFR domain | 115 | 0.774 | 0.955 (0.933, 0.977) | <0.0001 |
|  | Monthly migraine headache days by 50% | 115 | 0.821 | 0.942 (0.918, 0.967) | <0.0001 |
| *WPAI overall work productivity loss* | MSQ-RFP domain | 118 | 0.692 | 0.970 (0.953, 0.989) | 0.0015 |
|  | MSQ-RFR domain | 118 | 0.758 | 0.961 (0.941, 0.981) | 0.0002 |
|  | Monthly migraine headache days by 50% | 118 | 0.827 | 0.942 (0.918, 0.966) | <0.0001 |
| *WPAI non-work-related activity impairment* | MSQ-RFP domain | 183 | 0.703 | 0.972 (0.959, 0.985) | <0.0001 |
|  | MSQ-RFR domain | 183 | 0.809 | 0.952 (0.935, 0.968) | <0.0001 |
|  | Monthly migraine headache days by 50% | 183 | 0.735 | 0.967 (0.953, 0.982) | <0.0001 |

Logistic regression with selected anchor as a dependent variable and WPAI domain scores as independent variable was used to estimate the threshold value of WPAI presenteeism, overall productivity loss and non-work-related activity impairment changes from baseline to Month 3; This provided the greatest discriminative ability between the responder and non-responder groups. The Concordance (C) statistic was used to evaluate adequacy of model fitting through AUC of ROC curve analysis. The AUC varied between 0.5 (no difference by chance) and 1.0; higher values indicate a better discrimination ability for predictive model. C-values of 0.5 indicated no difference by chance, 0.7 to 0.8 showed acceptable discrimination, 0.8 to 0.9 indicated excellent discrimination, and ≥0.9 showed outstanding discrimination.

Abbreviations: AUC, area-under-curve; CI, confidence interval; CM, chronic migraine; EM, episodic migraine; MSQ, Migraine-Specific Quality of Life questionnaire; N, number of patients; OR, odds ratio; PGI-S, patient global impression of severity; RFP, Role Function-Preventive; RFR, role function-restrictive; ROC, receiver-operator characteristic; SE, standard error; WPAI, Work Productivity and Activity Impairment questionnaire.

**Supplementary Figure 1**. Spearman correlations of baseline WPAI domain scores with MSQ, and monthly migraine headache days

1. **Correlation of WPAI Scores with MSQ_RFP, MSQ_RFR, and Migraine Headache Days – All Patients**


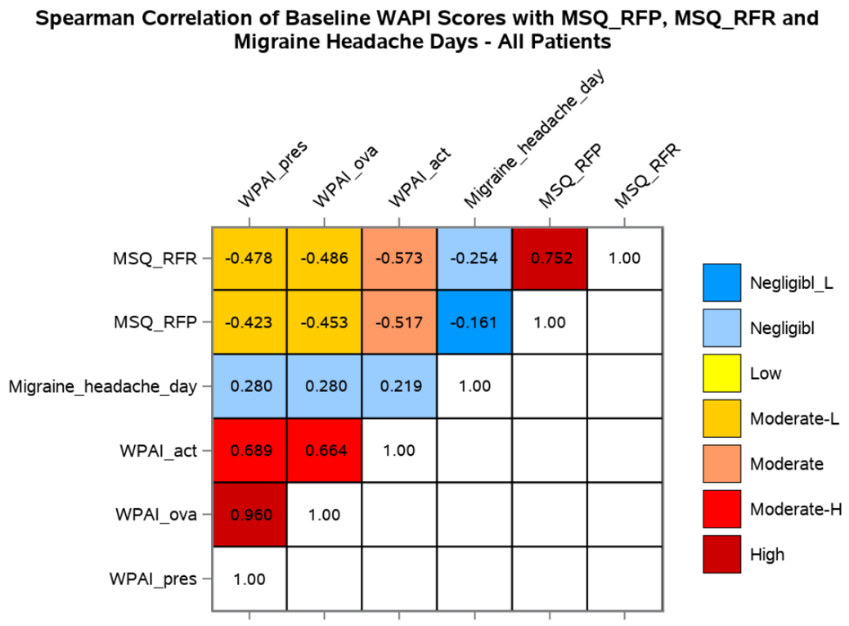


1. **Correlation of WPAI Scores with MSQ_RFP, MSQ_RFR, and Migraine Headache Days – EM Patients**


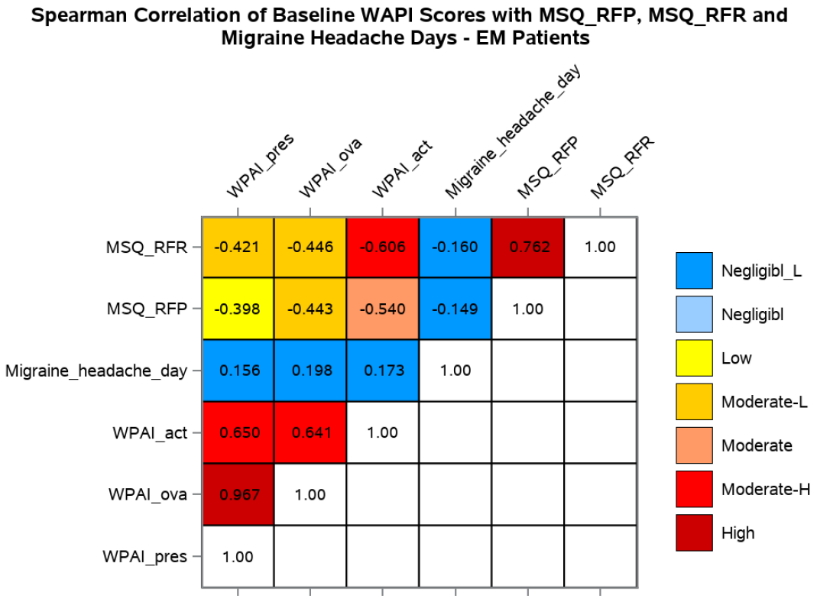


1. **Correlation of WPAI Scores with MSQ_RFP, MSQ_RFR, and Migraine Headache Days – All Patients**


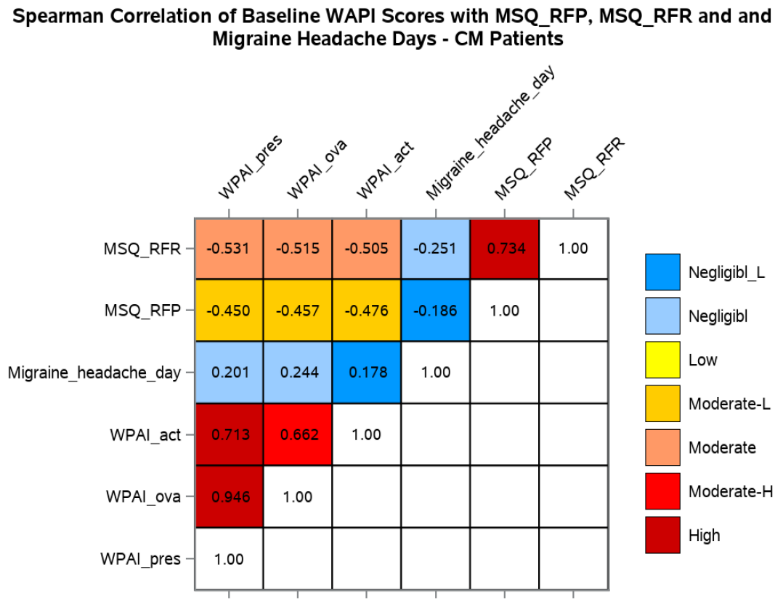


Negligible-L_: 0.0-0.2; Negligible: 0.2–0.3; Low: 0.3–0.4; Moderate-L: 0.4–0.5; Moderate: 0.5–0.6; Moderate-H: 0.6–0.7; High: 0.7–1

Abbreviations: MSQ, Migraine-Specific Quality of Life questionnaire; RFP, role function-preventive; RFR, role function-restrictive; WPAI, Work Productivity and Activity Impairment questionnaire; WPAI_act, WPAI non-work-related activity impairment; WPAI_ova, overall work productivity loss; WPAI_pres, WPAI presenteeism. Negligibl=Negligible

**Supplementary Figure 2.** Cumulative distribution function plots for change in WPAI from Baseline to Month 3

| **A. WPAI presenteeism and improvement of MSQ-RFP domain - Overall** | **B. WPAI presenteeism and improvement of MSQ-RFP domain - EM** |
| --- | --- |
| **** | **** |

| **C. WPAI presenteeism and improvement of MSQ-RFP domain - CM** | **D. WPAI presenteeism and improvement of MSQ-RFR domain - Overall** |
| --- | --- |
| **** | **** |

| **E. WPAI presenteeism and improvement of MSQ-RFR domain - EM** | **F. WPAI presenteeism and improvement of MSQ-RFR domain - CM** |
| --- | --- |
| **** | **** |

| **G. WPAI presenteeism and improvement of monthly headache days 50% - Overall** | **H. WPAI presenteeism and improvement of monthly headache days 50% - EM** |
| --- | --- |
| **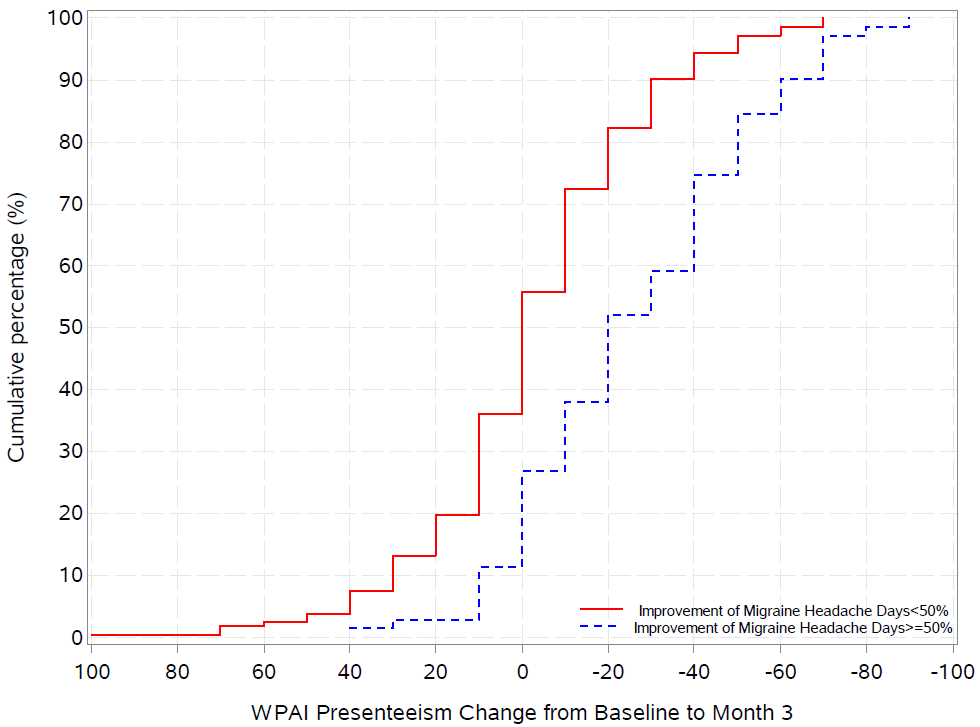** | **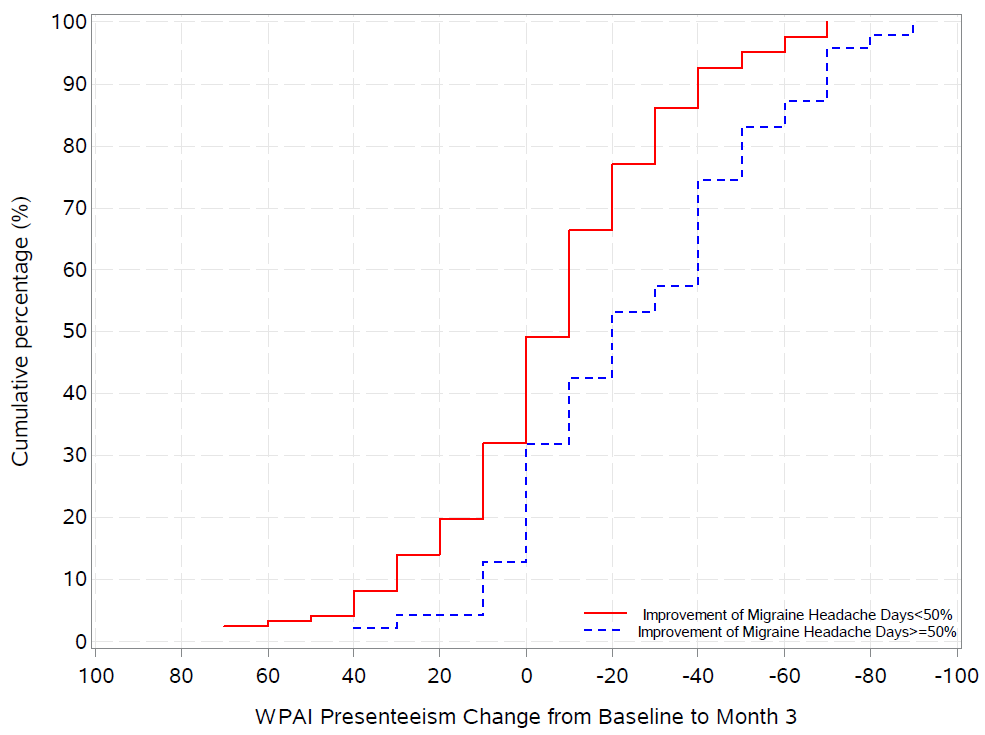** |

| **I. WPAI presenteeism and improvement of monthly headache days 50% - CM** |  |
| --- | --- |
| **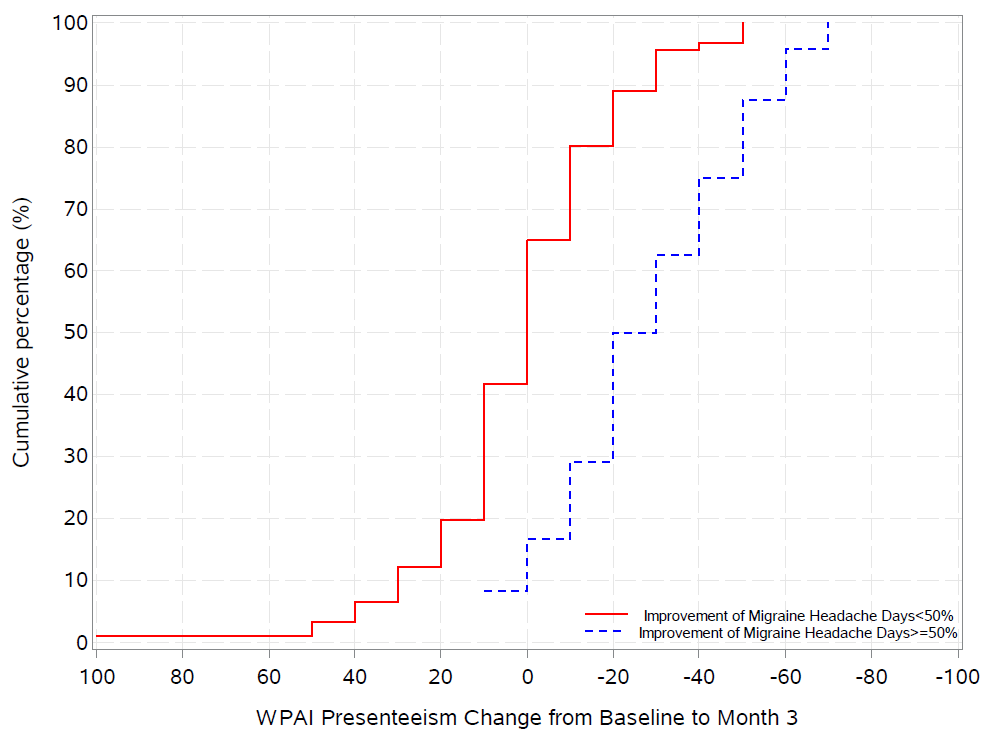** |  |

| **J. WPAI overall work productivity loss and improvement of migraine headache days 50% - Overall** | **K: WPAI overall work productivity loss and improvement of migraine headache days 50% - EM** |
| --- | --- |
| **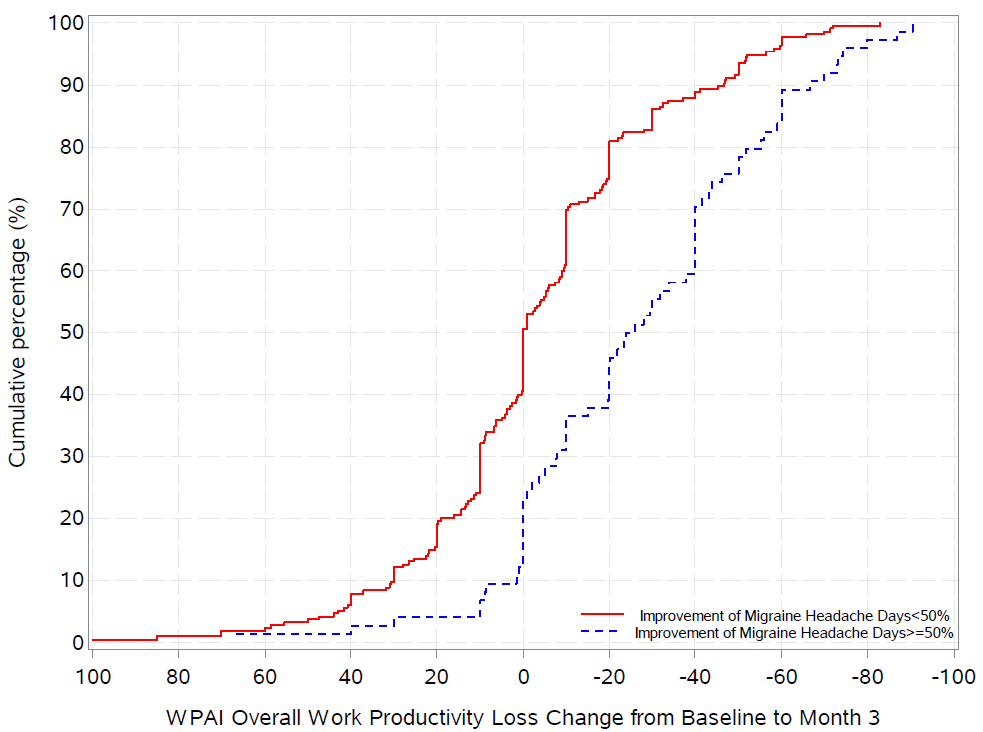** | **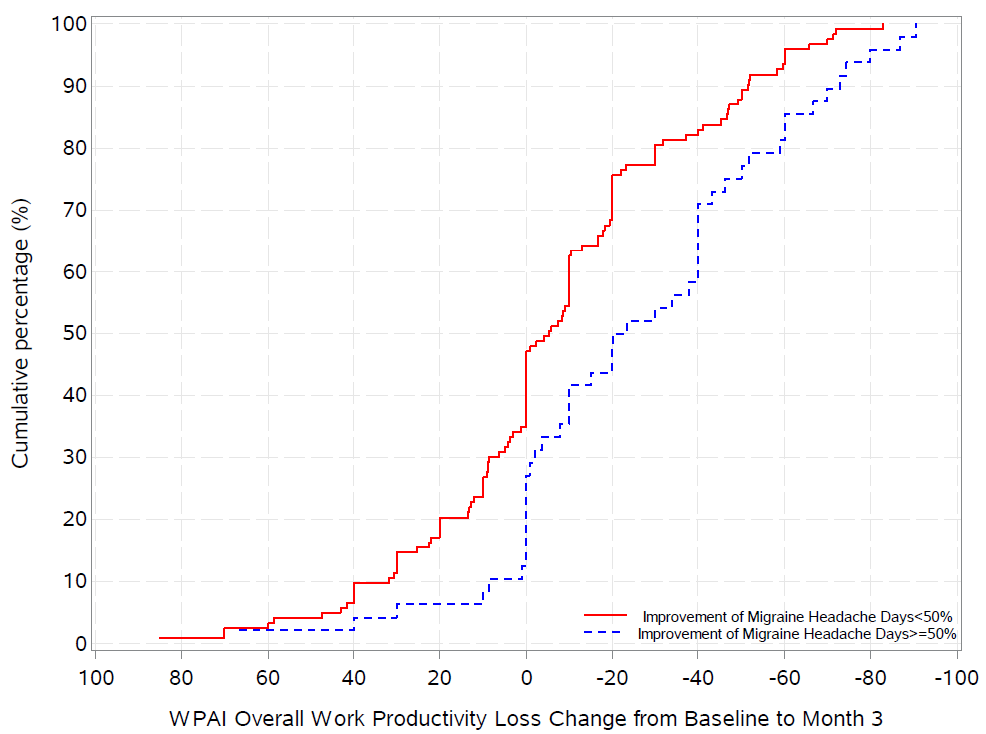** |

| **L. WPAI overall work productivity loss and improvement of migraine headache days 50% - CM** | **M: WPAI overall work productivity loss and improvement of MSQ-RFR - Overall** |
| --- | --- |
| **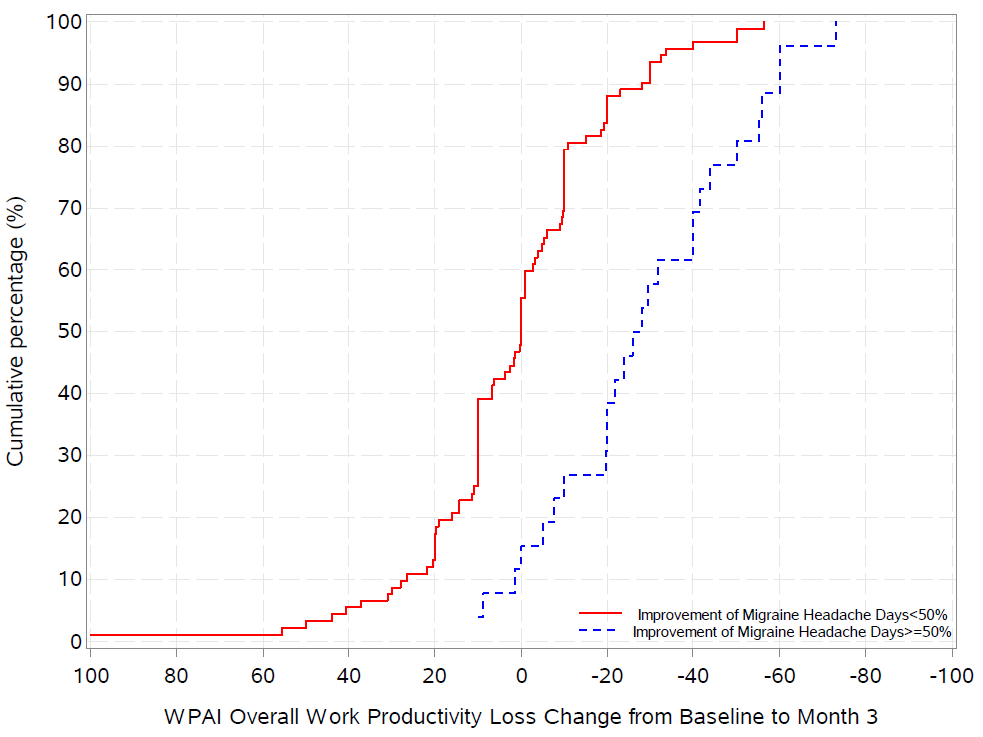** | **** |

| **N: WPAI overall work productivity loss and improvement of MSQ-RFR - EM** | **O: WPAI overall work productivity loss and improvement of MSQ-RFR - CM** |
| --- | --- |
| **** | **** |

| **P. WPAI overall work productivity loss and improvement of MSQ-RFP - Overall** | **Q: WPAI overall work productivity loss and improvement of MSQ-RFP - CM** |
| --- | --- |
| **** | **** |

| **R: WPAI non-work-related activity impairment and improvement of migraine headache days-50% - Overall**  **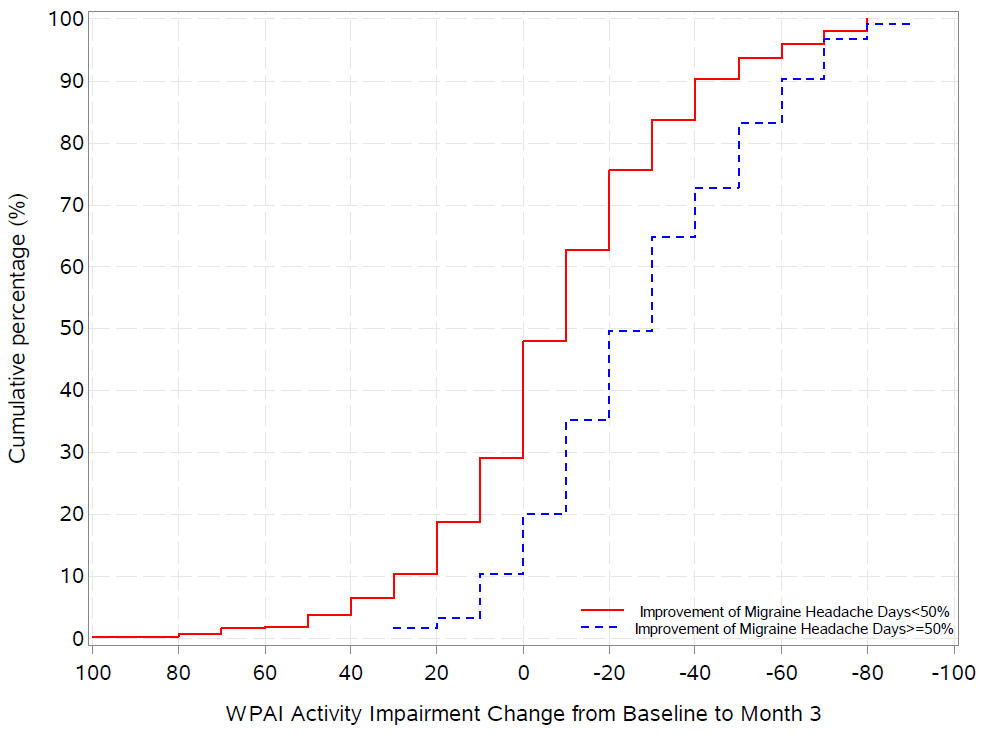** | **RS: WPAI non-work-related activity impairment and improvement of migraine headache days - 50% - EM**  **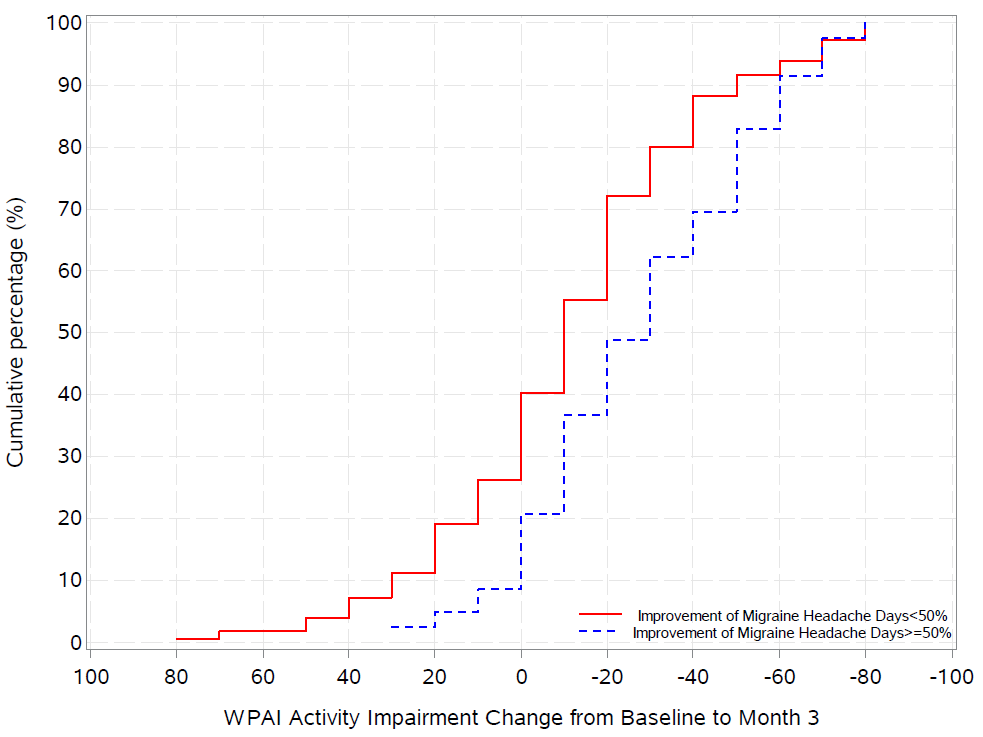** |
| --- | --- |
|  |  |
| **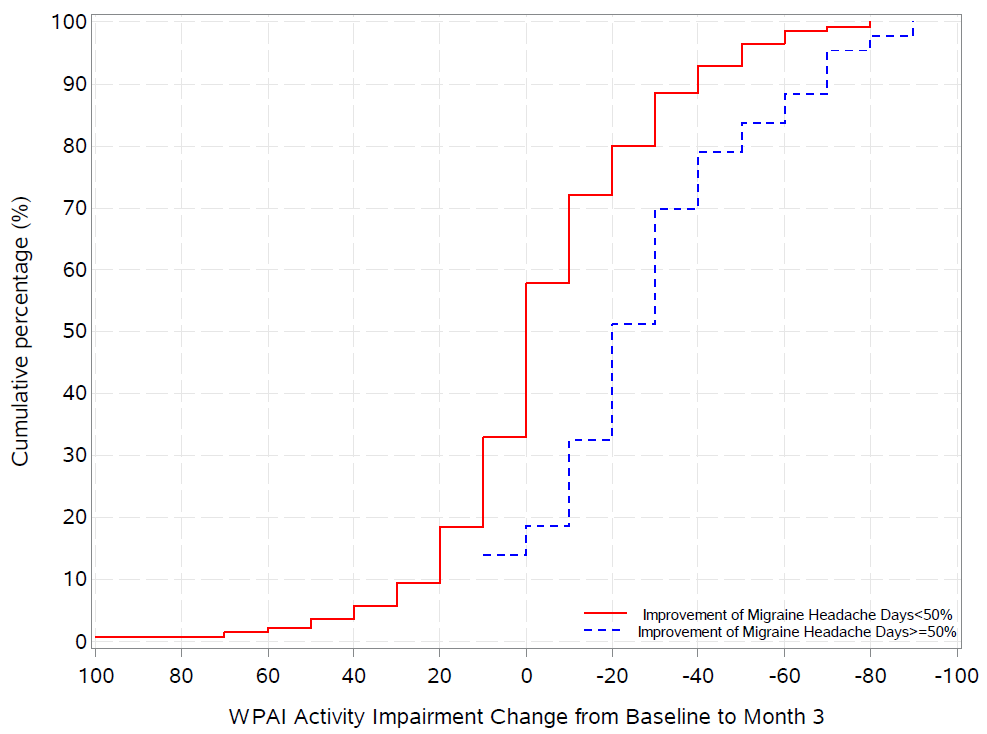T: WPAI non-work-related activity impairment and improvement of migraine headache days-50% - CM** |  |

WPAI scores are presented as % unit.

Abbreviations: CM, chronic migraine; EM, episodic migraine; MSQ, Migraine-Specific Quality of Life questionnaire; RFP, role function-preventive; WPAI, Work Productivity and Activity Impairment questionnaire.
